# Diabetes mellitus stimulates pancreatic cancer growth and epithelial-mesenchymal transition-mediated metastasis via a p38 MAPK pathway

**DOI:** 10.18632/oncotarget.9533

**Published:** 2016-05-21

**Authors:** Lishan Wang, Ying-Ying Bai, Yang Yang, Fangfang Hu, Yonghui Wang, Zeqian Yu, Zhangjun Cheng, Jiahua Zhou

**Affiliations:** ^1^ Department of General Surgery, Zhongda Hospital, School of Medicine, Southeast University, Nanjing, China; ^2^ Jiangsu Key Laboratory of Molecular and Functional Imaging, Department of Radiology, Zhongda Hospital, School of Medicine, Southeast University, Nanjing, China

**Keywords:** pancreatic cancer, diabetes mellitus, p38 MAPK, epithelial-mesenchymal transition, inflammation

## Abstract

Diabetes mellitus (DM) and its accompanying chronic inflammation promote tumor progression. p38 mitogen-activated protein kinase (MAPK) is an essential kinase for inflammation. The effects of p38 MAPK on epithelial-mesenchymal transition (EMT)-mediated diabetic pancreatic cancer metastasis remain unclear. Here, we demonstrate that p38 MAPK phosphorylation was significantly increased in pancreatic cancer cells treated with high glucose and in pancreatic tumors from diabetic animals. A p38 MAPK inhibitor significantly suppressed the proliferation and invasion of pancreatic cancer cells under high-glucose conditions. Moreover, p38 MAPK inhibition not only significantly decreased both the tumor volume monitored by magnetic resonance imaging and EMT-related metastasis but also increased the survival of diabetic mice bearing pancreatic tumors. Furthermore, the inflammation in diabetic animals bearing pancreatic tumors was also significantly lower after therapy. Collectively, our findings reveal that p38 MAPK inhibitors may provide a novel intervention strategy for diabetic pancreatic cancer treatment.

## INTRODUCTION

Pancreatic cancer (PC), one of the most fatal malignant diseases, is often diagnosed at later stages, and most treatments fail as a result of local recurrence and metastasis [1]. Diabetes mellitus (DM) serves not only as an early manifestation of PC but also as an independent risk factor for PC progression [2]. However, a mechanistic understanding of the high proliferative and metastatic potential of diabetic PC remains incomplete, and novel biomarkers or targets must still be identified to diagnose and treat PC.

The role of the epithelial-mesenchymal transition (EMT) in tumor metastasis has received much attention in recent years. EMT is a multifaceted process critical for the acquisition of a migratory, invasive and pluripotent stem cell-like phenotype and thus plays an important role in the metastatic process [3]. Several studies have demonstrated that a high glucose level induces tumor cell migration and invasion by stimulating EMT. Masur et al. reported that high glucose downregulates the level of E-cadherin (an epithelial cell marker) and enhances the activity of the protein kinase C-alpha pathway, thereby increasing the invasive potential of tumor cells [4].

DM is usually accompanied by chronic inflammation and oxidative stress. Several studies have indicated that the DM-related effects on the development of PC may be mediated by the associated inflammation and oxidative stress [5]. p38 mitogen-activated protein kinase (MAPK), as an essential kinase for inflammation, stimulates tumor growth. Studies have shown that the p38 MAPK signaling pathway is involved in high glucose-induced EMT in cultured renal tubular epithelial cells [6]. Therefore, we hypothesized that the phosphorylation of p38 MAPK could be associated with the EMT-mediated metastasis of diabetic PC and aimed to investigate the effects of p38 MAPK inhibitor-based therapy on pancreatic cancer.

## RESULTS

### p38 MAPK phosphorylation of PC cells in high-glucose (HG) environments *in vitro*

**Figure 1:**
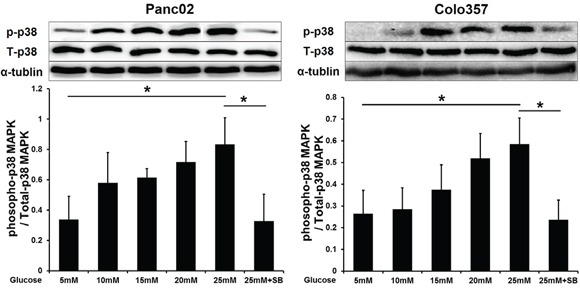
SB203580 inhibits the p38 MAPK phosphorylation induced by HG in PC cells Phosphorylation of p38 MAPK in PC cells cultured in media with varying concentrations of glucose (5-25 mM) or in high-glucose medium (25 mM) containing 10 μM SB203580 for 24 h (n=3/group). * *P* < 0.05.

We first explored the influence of HG on p38 MAPK activity by Western blot analysis. The phosphorylation level of p38 MAPK increased gradually in PC cells exposed to increasing concentrations of glucose (5-25 mM). Additionally, the p38 MAPK inhibitor SB203580 (10 μM) significantly inhibited p38 MAPK phosphorylation under the HG condition (Figure 1).

### p38 MAPK inhibition decreased both HG-induced proliferation and an anti-apoptosis effect in PC cells *in vitro*

**Figure 2:**
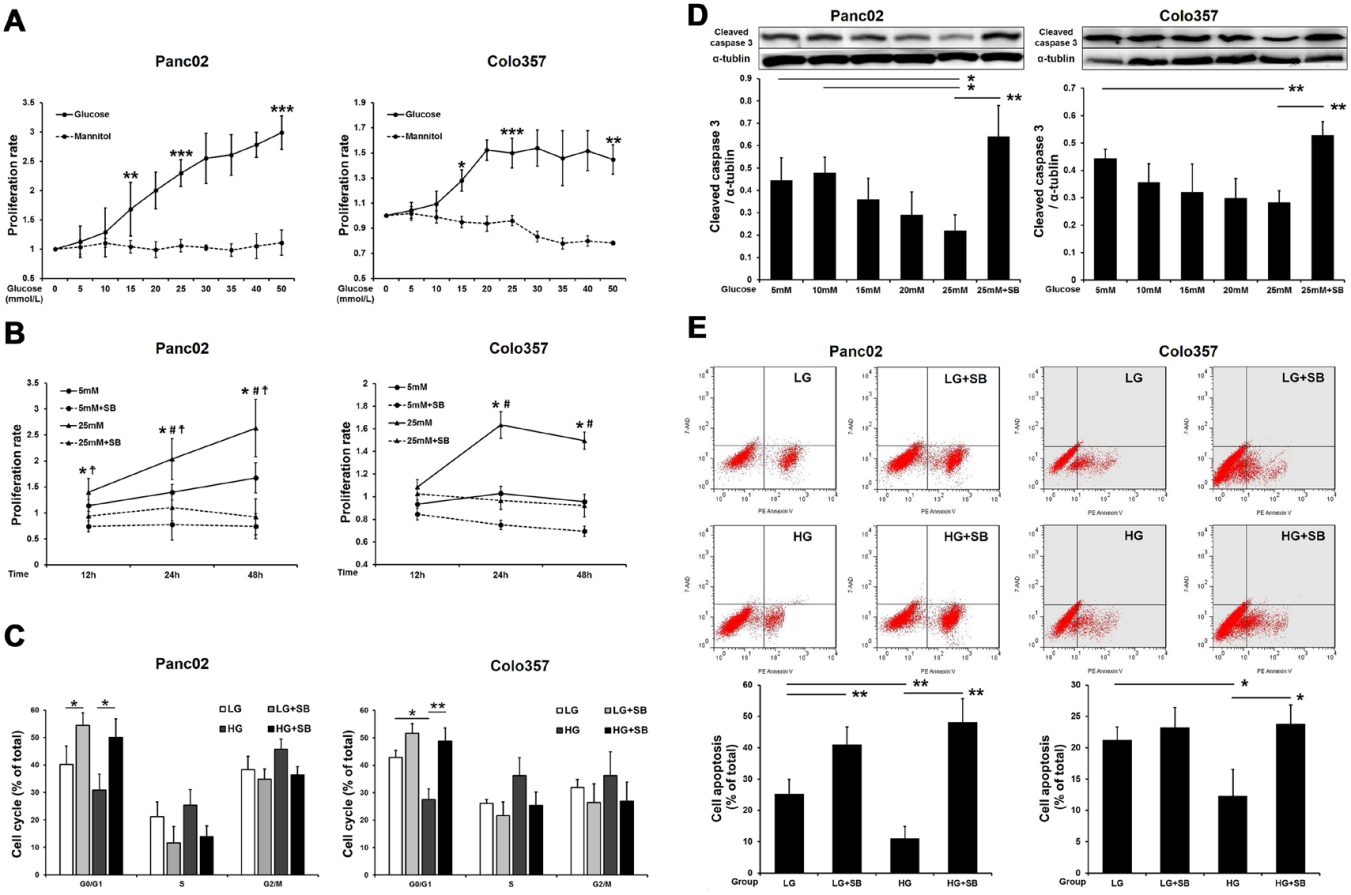
SB203580 inhibits HG-induced PC cell proliferation and anti-apoptotic effects *in vitro* **A.** Examination of the proliferative capacity of PC cells after cultivation in growth media containing varying concentrations of glucose or mannitol (0-50 mM) for 24 h (n=6/group). **B.** Examination of PC cell proliferation after cultivation in growth media for 12, 24 or 48 h (n=6/group). *, 5 mM vs. 5 mM plus SB203580; #, 5 mM vs. 25 mM; ‡, 25 mM vs. 25 mM plus SB203580. **C.** Flow cytometric analysis of the PC cell cycle at 24 h. **D.** Cleaved caspase 3 levels in PC cells after culture with different glucose concentrations for 24 h (n=3/group). **E.** Flow cytometric analysis of PC cell apoptosis at 24 h. * *P* < 0.05, ** *P* < 0.01, *** *P* < 0.001, # *P* < 0.05, and ‡ *P* < 0.05.

Next, MTT assays were used to determine the effect of glucose on PC cell proliferation. The results showed that HG stimulated the proliferation of PC cells in a concentration- (Figure 2A) and time-dependent manner (Figure 2B). In contrast, mannitol had no effect on tumor cell proliferation, indicating that this process was not affected by the increased osmotic pressure in the HG environment. The addition of SB203580 to low-glucose medium (5 mM) or HG medium (25 mM) resulted in a significant decrease in the proliferative capability of the tumor cells (Figure 2B). The tumor cell cycle progression was also investigated by flow cytometry. HG reduced the PC cell number in G_0_/G_1_; however, SB203580 extended the phases of S and G_2_/M after 24 h (Figure 2C), indicating that p38 MAPK inhibition suppressed HG-induced tumor cell growth.

The growth rate of tumor cells is determined by both cell proliferation and apoptosis. To investigate the effect of HG on the apoptosis of tumor cells, the levels of cleaved caspase 3 were measured by Western blot. The results showed that as the concentration of glucose increased, the apoptosis of tumor cells significantly decreased; however, SB203580 induced significant apoptosis in tumor cells cultured in an HG environment (Figure 2D). Similar results were also found for the cell apoptosis detected by flow cytometry at 24 h (Figure 2E).

### Effect of p38 MAPK inhibition on EMT in tumor cells cultured under HG conditions *in vitro*

**Figure 3:**
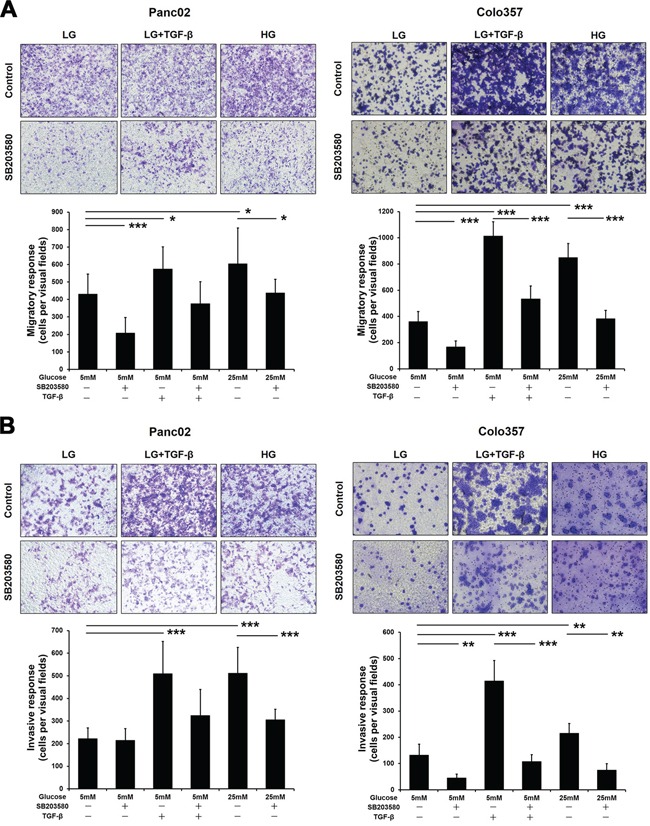
SB203580 inhibits HG-induced PC cell migration and invasion *in vitro* **A.** PC cells were seeded into the upper chambers of Transwell plates under serum-free conditions and cultured for 8 h. **B.** PC cells were seeded into Matrigel-coated upper chambers of Transwell plates and cultured for 24 h. The number of stained cells was counted in 10 randomly selected fields of view with a microscope at 200× magnification. * *P* < 0.05, ** *P* < 0.01, and *** *P* < 0.001.

EMT gives rise to tumor cell invasion and migration. We first analyzed the effects of HG and the p38 MAPK inhibitor on the invasive and migratory capabilities of PC cells using Boyden chambers. As a classic inducer of EMT, TGF-β was employed as the positive control. Both TGF-β and HG significantly enhanced the migratory and invasive capacities of PC cells (Figure 3A–3B). With SB203580 treatment, the migratory capabilities of the tumor cells cultured in both the LG and HG environments were markedly reduced (Figure 3A). However, treatment with SB203580 only reduced the invasive capability of Panc02 cells cultured in an HG environment (Figure 3B, left panel).

**Figure 4:**
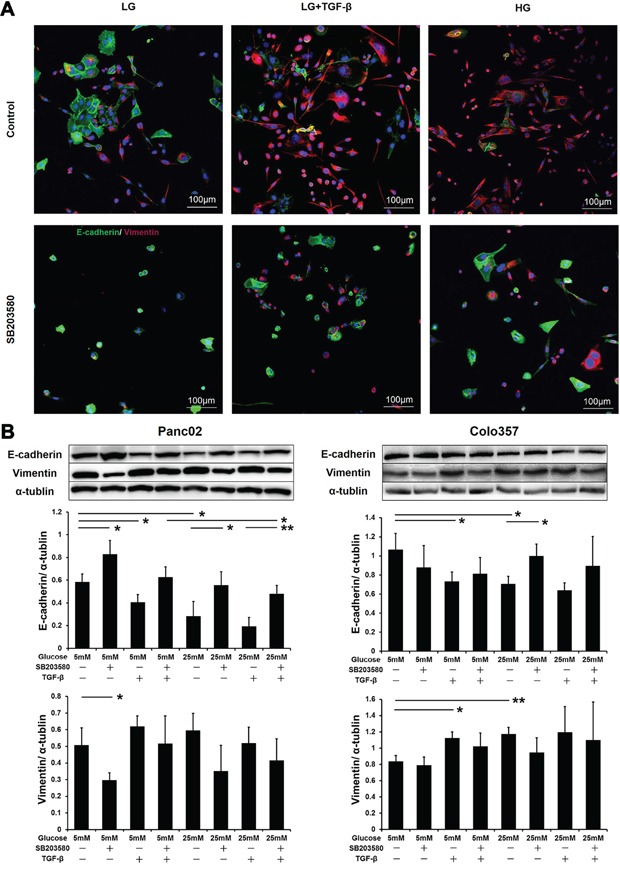
SB203580 inhibits HG-induced tumor cell EMT *in vitro* **A.** Panc02 cells were subjected to E-cadherin/vimentin double immunostaining. 4′,6-Diamidino-2-phenylindole (DAPI) was applied as an internal control. **B.** Expression levels of E-cadherin and vimentin in PC cells after culture in low-glucose medium, high-glucose medium, or medium containing 10 μM SB203580 or 10 ng/ml TGF-β for 24 h (n=3/group). * *P* < 0.05 and ** *P* < 0.01.

After incubation in an HG environment for 24 h, both Panc02 and Colo357 cells showed EMT phenotypes, with elongated morphology and dispersal from parental cells (Supplementary Figure S1A). To explore the role of p38 MAPK in EMT, the expression of E-cadherin and vimentin in Panc02 cells was examined. Dual immunofluorescence assays showed that both TGF-β and HG reduced E-cadherin expression and increased vimentin expression, thereby inducing EMT in Panc02 cells (Figure 4A). Western blot analyses confirmed the effects of these treatments on the E-cadherin and vimentin expression in PC cells (Figure 4B).

These experiments demonstrated that HG and TGF-β exerted similar EMT-stimulating effects on PC cells. Therefore, we examined the expression level of TGF-β in tumor cells under HG stimulation. Western blot results showed that HG did indeed upregulate TGF-β in tumor cells (Supplementary Figure S1B). These results indicate that HG levels might induce EMT in PC cells by stimulating TGF-β expression.

### Inflammatory factors involved in HG-induced EMT *in vitro*

**Figure 5:**
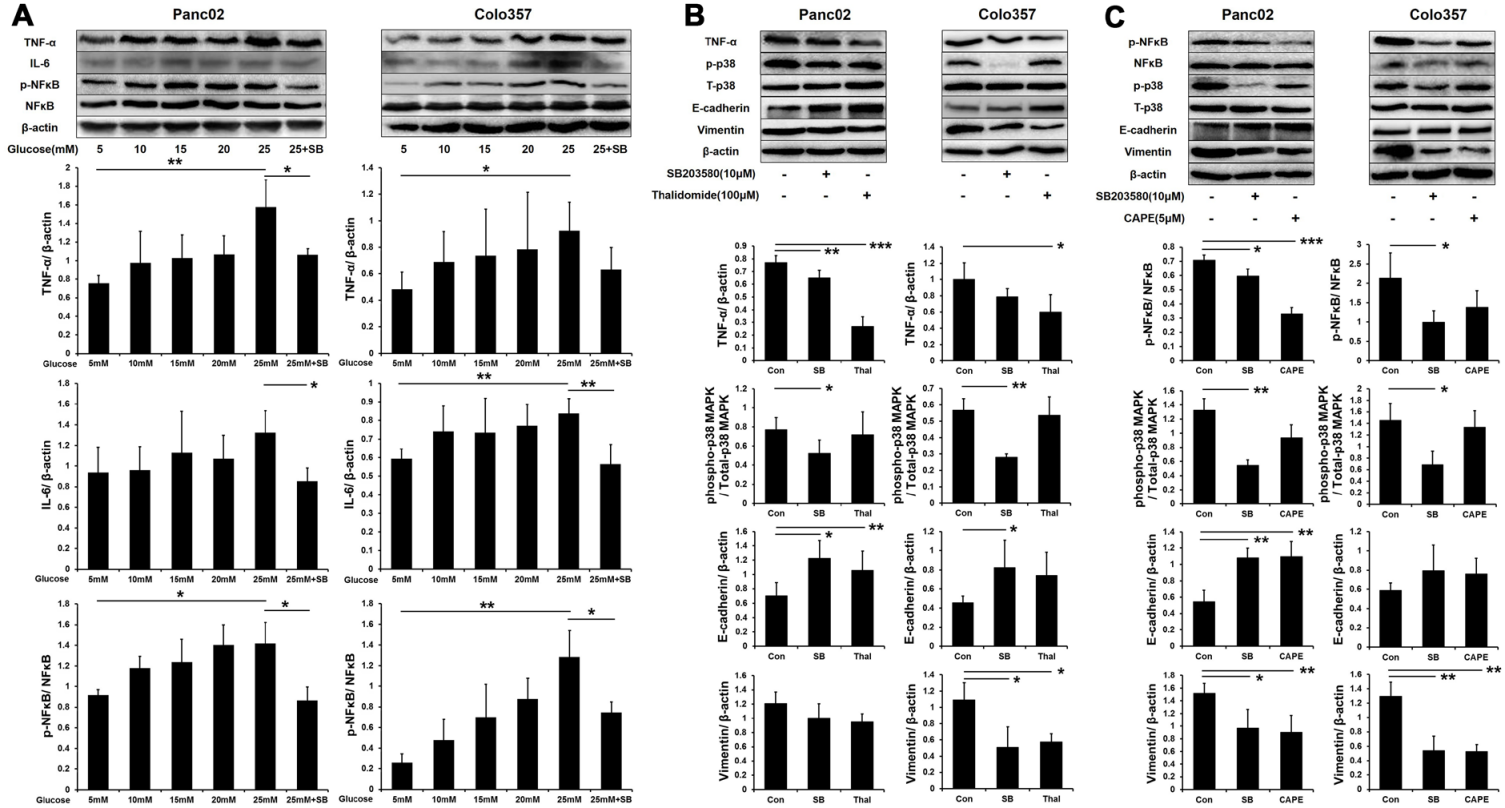
SB203580 inhibits HG-induced EMT through the TNF-α and p-NFκB pathways **A.** Expression level of TNF-α, IL-6 and p-NFκB in PC cells cultured in media with varying concentrations of glucose (5-25 mM) or in HG medium (25 mM) containing 10 μM SB203580 for 24 h (n=3/group). **B.** PC cells were treated with 100 μM thalidomide for 24 h, and EMT markers were detected (n=3/group). **C.** PC cells were treated with 5 μM CAPE for 24 h, and EMT markers were detected (n=3/group). * *P* < 0.05, ** *P* < 0.01, and *** *P* < 0.001.

Hyperglycemia triggers chronic systemic inflammatory responses and induces the expression of a variety of inflammatory factors, including NFκB, TNF-α and IL-6, which also promote PC progression. Therefore, we examined the expression of inflammatory factors under the HG condition *in vitro*. The expression of TNF-α, IL-6 and phospho-NFκB (p-NFκB) gradually increased in PC cells that were treated with increasing concentrations of glucose (5-25 mM) (Figure 5A). Meanwhile, SB203580 significantly suppressed the expression of TNF-α, IL-6 and p-NFκB under the HG condition *in vitro*, indicating that HG induced inflammatory factor activation through the p38 MAPK pathway.

Furthermore, we investigated the relationship between inflammatory factors and EMT in PC cells. To suppress inflammation *in vitro*, 100 μM thalidomide (a TNF-α inhibitor) and 5 μM CAPE (an NFκB inhibitor) were employed. Western blot showed that the expression of TNF-α was inhibited by both thalidomide and SB203580 in PC cells (Figure 5B). Thalidomide and SB203580 both increased the expression of E-cadherin in PC cells but decreased the expression of vimentin in Colo357 cells only. CAPE significantly decreased the phosphorylation of NFκB in PC cells (Figure 5C). Moreover, CAPE also increased the E-cadherin expression in Panc02 cells and decreased the vimentin expression in both cell lines. These results indicated that p38 MAPK induced EMT progression through increasing the expression of TNF-α and p-NFκB.

### Effects of SB203580 treatment on diabetic pancreatic cancer-bearing mice and tumor growth *in vivo*

**Figure 6:**
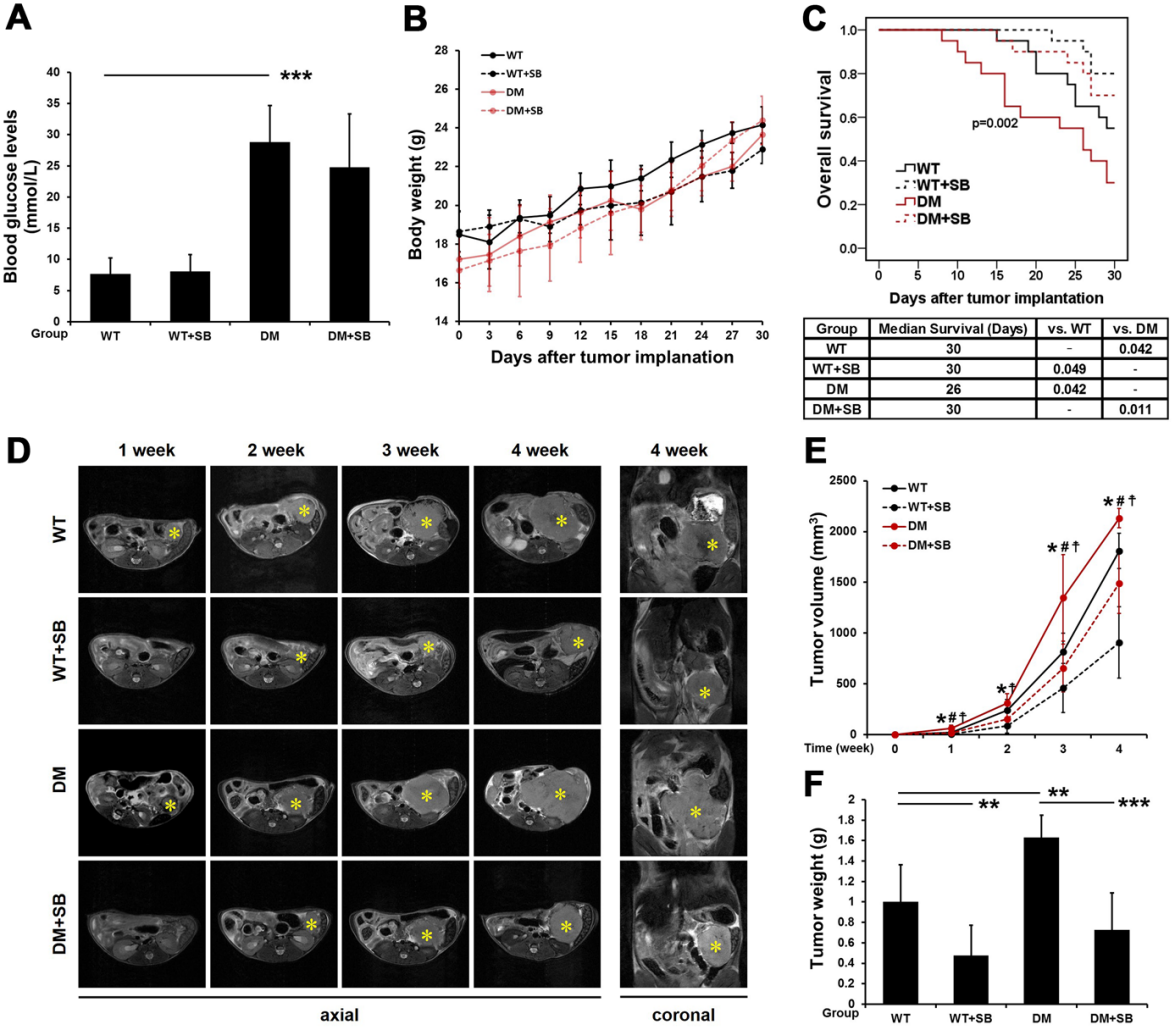
SB203580 significantly inhibits tumor growth and improves the prognosis of diabetic mice with pancreatic cancer **A.** Examination of blood glucose levels in the different groups (n=20/group). **B.** All mice were weighed every 3 days after tumor transplantation. **C.** Survival curves of the different groups of mice over a period of up to 30 days immediately following tumor transplantation. **D.** Tumors in the different groups of mice were subjected to T_2_ WI scans 1, 2, 3 and 4 weeks after tumor transplantation. * indicates the location of the primary pancreatic tumor. **E.** The volumes of the tumors in the different groups at different time points were quantitatively analyzed based on the MRI data (n=6-10/group). *, WT vs. WT plus SB203580; #, WT vs. DM; and ‡, DM vs. DM plus SB203580. **F.** Quantitative analysis of the weight of pancreatic tumors in the different groups 4 weeks after tumor transplantation (n=6-10/group). WT: wild type; DM: diabetes mellitus. * *P* < 0.05, ** *P* < 0.01, *** *P* < 0.001, # *P* < 0.05, and ‡ *P* < 0.05.

A mouse model of diabetic pancreatic cancer was established in the present study. After 7 days of continuous SB203580 treatment, blood glucose levels were examined. The blood glucose levels in the DM group were significantly higher than those in the wild type (WT) group. The therapeutic intervention with SB203580 had no effect on the blood glucose levels (Figure 6A). Neither diabetes nor SB203580 treatment had a significant impact on body weight in any of the groups (Figure 6B).

The 30-day survival was assessed by Kaplan-Meier survival analysis. The prognosis of pancreatic cancer-bearing mice in the DM group was poorer than that in the WT group. SB203580 treatment significantly improved the prognosis of mice with pancreatic cancer, with or without diabetes (Figure 6C).

### Dynamic monitoring of the effect of SB203580 treatment on the volume of pancreatic tumors *in vivo* using magnetic resonance imaging (MRI)

The pancreatic cancer group (WT), the SB203580-treated pancreatic cancer group (WT+SB), the concurrent diabetes and pancreatic cancer group (DM), and the SB203580-treated diabetic pancreatic cancer group (DM+SB) were subjected to MR scans 1, 2, 3 and 4 weeks after tumor transplantation. Figure 6D shows axial MR slices of the tumors along the largest dimension in the different groups of mice at various time points, and the right panels show the coronal MR slices of the tumors along the largest dimension in the 4^th^ week. Changes in tumor volume were calculated for each group of mice using ImageJ software. The volumes of the pancreatic tumors in the DM group were significantly larger than those in the WT group. Tumor volumes were reduced upon therapeutic intervention with SB203580 in the WT+SB and DM+SB groups at all the time points examined (Figure 6E).

After the 4^th^ week MR scan, all the mice were sacrificed, and the orthotopic pancreatic tumors were dissected and weighed. The tumor weight in the diabetic group was significantly higher than that in the non-diabetic group. SB203580 treatment markedly reduced the tumor weight (Figure 6F). Based on the tumor volume and weight measurements, it can be concluded that SB203580 treatment significantly inhibited diabetes-associated tumor growth.

### Effect of SB203580-based therapeutic intervention on pancreatic cancer metastasis in diabetic mice

**Figure 7:**
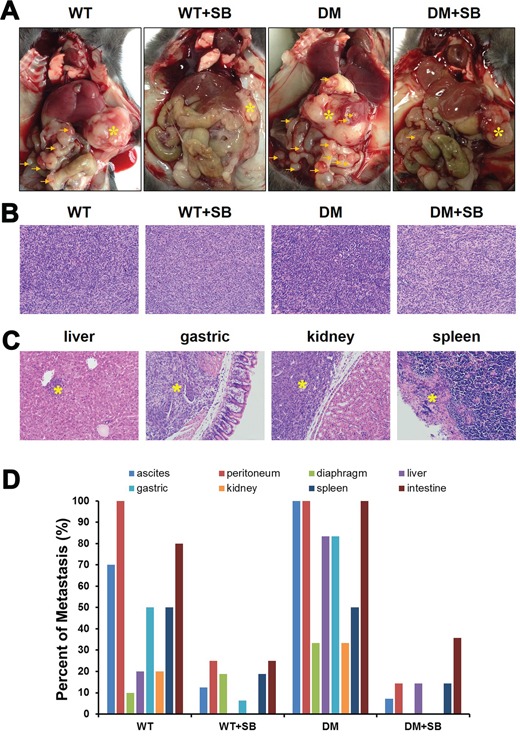
Treatment with SB203580 reduces the incidence of cancer metastases in diabetic mice with pancreatic cancer **A.** Representative gross anatomy images of the different groups of mice. * marks the location of the primary pancreatic tumors, while the arrows indicate the metastatic sites in the peritoneal cavity. **B.** Representative HE-stained images of orthotopic pancreatic tumors in the different groups of mice. Magnification, 200×. **C.** Representative HE-stained sections of the potential target organs of pancreatic tumor metastasis. Magnification, 200×. **D.** Examination of the presence or absence of ascites and ratios of peritoneal, diaphragmatic, liver, gastric, renal, splenic and small bowel metastases.

Metastasis is the most important factor affecting prognosis. However, the influence of diabetes on pancreatic cancer metastasis is rarely reported. In the present study, all the mice were subjected to an exploratory laparotomy 4 weeks after the tumor transplantation. Both the tumor size and the incidence of abdominal metastasis in the DM group were higher than those in the WT group. Treatment with SB203580 reduced the tumor volume and the incidence of abdominal metastasis (Figure 7A).

Hematoxylin and eosin (HE) staining was performed to examine the size of orthotopic pancreatic tumors in the different groups (Figure 7B) and the metastasis to various organs (liver, stomach, kidney and spleen) (Figure 7C). A detailed exploratory laparotomy was combined with microscopic observations of HE-stained organ sections to determine the presence of pancreatic cancer-induced ascites and the presence of peritoneal, diaphragmatic, liver, gastric, renal, splenic and small bowel metastases in each mouse. The metastatic ratio was quantified for each group of mice. The results showed that the metastatic frequency in the diabetic group was higher than that in the non-diabetic group. In addition, SB203580-based therapeutic intervention reduced the incidence of cancer metastasis (Figure 7D).

### Effect of SB203580 treatment on EMT occurrence and on the expression of inflammatory factors in primary lesions and metastatic lesions in diabetic mice with pancreatic cancer

**Figure 8:**
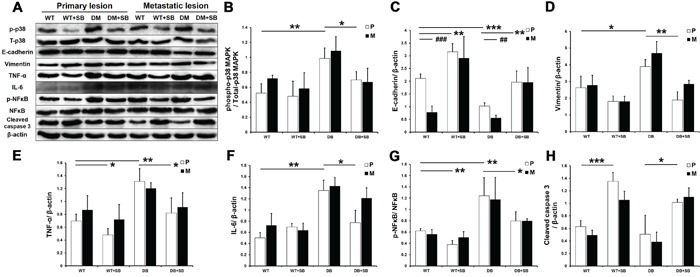
SB203580 inhibits EMT and the expression of inflammatory factors in diabetic pancreatic cancer *in vivo* **A.** p38 MAPK phosphorylation and the protein expression levels of E-cadherin, vimentin, TNF-a, IL-6, p-NFκB, NFκB and cleaved caspase 3 in primary tumor tissues and metastatic lesions (intestinal metastatic sites) obtained from the WT, WT+SB, DM and DM+SB groups. **B-H.** Quantitation of the expression levels of the above proteins in the primary tumors from the different groups (n=5/group). P: primary lesion; M: metastatic lesion. * *P* < 0.05, ** *P* < 0.01, *** *P* < 0.001, ## *P* < 0.01, and ### *P* < 0.001.

The phosphorylation of p38 MAPK was analyzed using Western blot for the different groups 4 weeks after tumor transplantation. The phosphorylation of p38 MAPK was significantly elevated in the diabetic mice and inhibited by SB203580 in the DM+SB group (Figure 8A and 8B).

The effect of SB203580 on the tumor EMT was further examined *in vivo*. Western blot analysis showed that the E-cadherin expression was markedly downregulated in the diabetic group compared with the non-diabetic group, whereas the vimentin expression was not significantly affected (Figure 8C and 8D). SB203580 significantly upregulated E-cadherin in the diabetic group and downregulated vimentin in both the WT+SB and DM+SB groups. Furthermore, E-cadherin expression was significantly lower in metastatic lesions compared to primary lesions without SB203580 treatment (Figure 8C), indicating that the cells in metastatic lesions have a greater invasive potential. These results demonstrated that SB203580 is capable of suppressing the tumor EMT induced by HG levels *in vivo*.

Next, we examined the expression of inflammatory factors in the mouse tumors (Figure 8E–8G). Western blot results showed that diabetes resulted in significantly elevated TNF-α, IL-6 and p-NFκB expression in primary and metastatic tumors, while SB203580 treatment markedly reduced their expression (Figure 8E–8G). Immunohistochemical assays produced similar results in primary lesions (Supplementary Figure S2).

SB203580 also significantly upregulated the cleaved caspase 3 levels *in vivo* (Figure 8H). An immunohistochemical analysis of Ki-67 further showed that SB203580 inhibited diabetes-induced cell proliferation (Supplementary Figure S2).

## DISCUSSION

Hyperglycemia in diabetic patients promotes the growth and metastasis of tumor cells [7]. The identification of effective therapeutic targets may facilitate standardized regimens for the clinical treatment of patients with pancreatic cancer complicated by diabetes. The present study showed that HG increased p38 MAPK phosphorylation in pancreatic cancer cells while simultaneously enhancing the proliferative, anti-apoptotic, invasive and migratory capabilities of the cells. The p38 MAPK inhibitor suppressed the HG-induced proliferation, invasion, migration and EMT of tumor cells. In addition, an SB203580-based therapeutic intervention significantly reduced the growth and metastasis of orthotopic pancreatic tumors *in vivo*. These results demonstrate that HG was capable of inducing tumor growth and metastasis and that these effects were closely related to the p38 MAPK signaling pathway.

p38 MAPK is mainly involved in cellular stress reactions and inflammatory responses [8]. In addition, p38 MAPK regulates the proliferation, apoptosis and metastasis of tumor cells [9]. In colon cancer, p38 MAPK is necessary for the maintenance of active tumor cell growth [10]. Application of the p38 MAPK inhibitor SB203580 significantly increased the sensitivity of colon cancer cells to the killing effects of exisulind [11]. In myelomas, p38 MAPK inhibitors diminished IL-6- and VEGF-mediated paracrine effects, thereby inhibiting the proliferation of tumor cells [12]. Moreover, studies of nephropathy and peritoneal fibrosis in the context of diabetes have revealed that high blood glucose induces EMT in epithelial cells through the p38 MAPK signaling pathway, thereby promoting disease progression [13, 14]. EMT is crucial not only for fibrotic diseases. After the loss of the epithelial phenotype and the transition into the mesenchymal state, tumor cells acquire enhanced migratory, invasive, anti-apoptotic and extracellular matrix (ECM)-degrading capabilities [15]. Therefore, EMT occurs during the process by which tumors spread to distant sites. We hypothesized that in the case of hyperglycemia complicated by neoplastic diseases, disease progression would be related to p38 MAPK signaling, including tumor cell proliferation and the EMT process. We found that HG promoted proliferation and enhanced invasiveness in pancreatic cancer cells, which was consistent with previous results [16, 17]. In addition, we discovered that HG induced p38 MAPK phosphorylation in pancreatic cancer cells, both *in vitro* and *in vivo*. Inhibition of p38 MAPK significantly decreased the tumor volume and markedly reduced EMT. Our results thus indicate that in diabetic conditions, the proliferation and metastasis of pancreatic cancer cells are induced by p38 MAPK signaling, a connection that has not been previously reported.

In recent years, chronic pancreatitis has been recognized as a central event in the development and progression of pancreatic cancer [18]. Inflammation-induced activation of STAT3 and NFκB promotes the survival, proliferation and EMT of tumor cells, thus contributing to the development and progression of pancreatic cancer [19, 20]. Diabetes-induced p38 MAPK activation promotes the production of inflammatory factors, including TNF-α, IL-1, IL-4, IL-6 and IL-8, by tissues and cells [21]. Our experiments showed that the presence of diabetes led to elevated expression of TNF-α, IL-6 and p-NFκB in tumor tissues. These inflammatory factors promote tumor growth, exert anti-apoptotic activities and induce EMT [22–24]. Figures 5 and 8 show that SB203580 significantly downregulated these inflammatory factors *in vitro* and *in vivo*, inhibited tumor growth and metastasis and significantly prolonged the survival of mice with pancreatic cancer. The above results demonstrate that p38 MAPK represents a key signaling pathway in the regulation of inflammatory factor levels in pancreatic cancer and that p38 MAPK inhibitors inhibit the progression of pancreatic cancer by reducing inflammatory responses.

Animal studies contribute greatly to our understanding of the malignant characteristics of tumors *in vivo*. In the present study, we used 7 tesla MRI to monitor the growth of orthotopically transplanted pancreatic cancer tumors in mice with concurrent diabetes. The traditional caliper-based examination method is unable to assess tumor growth and metastasis dynamically. Due to the irregular growth of tumors and the varying degrees of anatomical precision, the tumor size measurements were not very accurate. MRI, which does not exhibit these depth or angle limitations, allows accurate calculation of tumor volumes [25]. In the present study, the size of the pancreatic cancers was dynamically monitored *in vivo* by MRI for up to 4 weeks after the establishment of the model.

The present study has some limitations: 1) p38 MAPK inhibitors cannot specifically target tumor cells *in vivo*. Based on a literature review, we used SB203580 at a dose of 5 mg/kg body weight, which significantly suppressed p38 MAPK phosphorylation in cancer cells [26]. 2) We established an animal model of type 2 diabetes according to a procedure reported in the literature. Type 2 diabetes is usually accompanied by other metabolic abnormalities, including hyperlipidemia and hyperinsulinemia. We were unable to distinguish the effects on tumors induced by hyperglycemia, hyperlipidemia, hyperinsulinemia or combinations of these metabolic abnormalities. 3) Advanced pancreatic cancer metastasizes mainly to the liver. However, no significant liver metastases were detected in the present study by MRI or gross anatomy analysis. Only liver micrometastases were observed by HE staining. It is likely that the resolution of MRI was not sufficiently high to detect very small lesions, and the observation period was too brief to produce detectable lesions.

In summary, the present study demonstrates that a p38 MAPK inhibitor can suppress HG- or diabetes-induced tumor growth. In addition, the p38 MAPK inhibitor partially reversed EMT and inhibited distant metastasis by reducing inflammatory responses. Therefore, p38 MAPK is likely to become an effective therapeutic target for treating diabetic pancreatic cancer patients.

## MATERIALS AND METHODS

### Cell culture and reagent

Panc02 cells, derived from C57BL/6J mice, were kindly provided by Dr. M. Li (Baylor College of Medicine, Houston, Texas 77030, USA). The Colo357 cell line was provided by prof. Helmut Friess (Technical University Munich, Munich, Germany). PC cells were maintained in low glucose (5 mM; LG) Dulbecco's modified Eagle's medium (DMEM) supplemented with 10% fetal bovine serum (FBS), 100 U/ml penicillin and 100 μg/ml streptomycin and incubated at 37°C in 5% CO_2_. Various amounts of glucose were added to the culture medium to create cell growth environments containing different concentrations of glucose. In addition, 10 μM SB203580 (Sigma-Aldrich, St. Louis, MO, USA) or 10 ng/ml TGF-β (PeproTech, Rocky Hill, USA) was added to the culture medium. Caffeic acid phenethyl ester (CAPE) and thalidomide were purchased from Selleck Chemicals (Houston, TX, USA).

### Cell proliferation assay

MTT (3-(4,5-dimethylthiazol-2-yl)-diphenyltetrazolium bromide) was used to examine the proliferative capacity of PC cells. The cells were starved in serum-free medium for 24 h and then seeded at a density of 5,000 cells per well in culture media containing 5 to 50 mM glucose in the presence or absence of 10 μM SB203580. Cells cultured in media containing equal concentrations of mannitol served as the osmotic pressure control. After incubation at 37°C for 12, 24, or 48 h, 20 μl MTT solution (5 mg/ml) was added to each well, and the cells were incubated for another 4 h. Subsequently, 150 μl dimethyl sulfoxide (DMSO) was added to each well and incubated for 15 min. Finally, absorbance was measured at 490 nm in an enzyme-linked immunosorbent assay (ELISA) microplate reader (Bio Rad, CA, USA).

### Flow cytometry assay

To evaluate the effects of cell growth inhibition by SB203580 under the HG condition, the cell cycle and apoptosis were analyzed. After SB203580 treatment under HG or LG conditions for 24 h, 1 × 10^6^ PC cells were harvested. According to the reagent instructions, the cell cycle and apoptosis were, respectively, evaluated using PI/RNase Staining Buffer (BD Bioscience, Bedford, MA) and a PE Annexin V Apoptosis Detection Kit I (BD Bioscience, Bedford, MA). Flow cytometry was performed using a Cytomics FC 500 flow cytometer (Beckman Coulter). The cell cycle and cell apoptosis results were analyzed using FlowJo software (TreeStar, Ashland, OR, USA).

### Cell invasion and migration assays

The invasion and migration of pancreatic cancer cells were assessed by the Boyden chamber assay. In the invasion assay, the membranes of the upper chambers were coated with 50 μl Matrigel (BD Bioscience, Bedford, MA). The cells were cultured in LG medium (5 mM) or HG medium (25 mM) in the presence or absence of 10 μM SB203580 or 10 ng/ml TGF-β for 24 h. Next, 5 × 10^4^ cells were harvested, resuspended in serum-free culture medium and seeded into the upper chambers. The lower chambers were filled with medium containing 20% FBS (chemotactic factor). The cells were incubated in a 37°C incubator for 8 h (migration assay) or 24 h (invasion assay). Subsequently, the upper chambers were removed. The cells attached to the lower surface of the membranes were fixed in methanol, stained with crystal violet and photographed with an optical microscope.

### Animal model

Animal experiments abided by the Guidelines for Animal Care and Use issued by the Medical School of Southeast University Institutional Animal Care and Use Committee. Eighty male C57BL/6J mice, aged 4-6 weeks, were first randomly divided into 2 groups (WT group and DM group). Mice in the DM group were given an intraperitoneal injection of sterile citrate buffer containing streptozotocin (25 mg/kg, Sigma Aldrich, St. Louis, MO, USA), while mice in the WT group were injected intraperitoneally with the same volume of citrate buffer. After continuous administration of streptozotocin for 5 days, the DM group was fed a high-fat diet (58 kcal% fat w/sucrose, Research Diets) for 3 weeks to establish a model of type 2 diabetes. The WT group was given a normal diet. Orthotopic pancreatic cancer models were established simultaneously in both groups of mice. The detailed procedures for establishing orthotopic pancreatic cancer were described in our previous study [27]. After establishment of the model, the 2 groups of mice were each further randomly divided into 2 subgroups (WT and WT+SB; DM and DM+SB). The WT+SB and DM+SB groups received intraperitoneal injections of 5 mg/kg SB203580 daily for 7 consecutive days, whereas the WT and DM groups were injected with an equal volume of saline.

### MRI scan

The *in vivo* MRI assays were performed using a 7.0 T small animal MR scanner (Bruker PharmaScan, Ettlingen, Germany) and a 31-mm inner diameter transmit-receive quadrature volume coil. Each mouse was subjected to a dynamic T_2_-weighted imaging (WI) scan 1, 2, 3 and 4 weeks after tumor transplantation to monitor tumorigenesis. The tumor growth curves were drawn according to the MR data in the four groups. T_2_ WI images were acquired with a respiratory-gated fast spin echo sequence, with a repetition time of 3,000 msec, an echo time of 36 msec, a field of view of 30 × 30 mm, a matrix of 256 × 256, 24 slices at 1 mm per slice, and an acquisition time of approximate 8 min.

### Western blot analysis

Cells or tumor tissues were collected from each group of mice. Protein concentrations were determined using the bicinchoninic acid (BCA) assay. The proteins (40 μg/sample) were separated by sodium dodecyl sulfate polyacrylamide gel electrophoresis (SDS-PAGE) and then transferred to polyvinyl difluoride (PVDF) membranes. The blots were incubated with rabbit anti-mouse primary antibodies at 4°C overnight in a buffer containing 5% skim milk and then with a horseradish peroxidase (HRP)-conjugated goat anti-rabbit secondary antibody at 37°C for 2 h. Primary antibodies against p38 MAPK, NFκB, phospho-p38 MAPK and phospho-NFκB were purchased from Cell Signaling Technology (Beverly, MA, USA). The mouse E-cadherin antibody was purchased from R&D systems (Minneapolis, MN, USA). Primary antibodies against vimentin, IL-6, TNF-α, cleaved caspase 3 and human E-cadherin were obtained from Abcam (Cambridge, MA, USA). Protein levels (band densities) were analyzed quantitatively using ImageJ software (NIH, USA).

### Immunofluorescence and immunohistochemistry staining

Cells were treated under LG or HG culture conditions with 10 μM SB203580 or 10 ng/ml TGF-β for 24 h. Subsequently, the cells were fixed in 4% paraformaldehyde for 15 min, blocked with phosphate-buffered saline (PBS) containing 0.1% Triton and 10% serum for 2 h and then incubated with polyclonal sheep anti-mouse E-cadherin antibodies (10 μg/ml) and a monoclonal rabbit anti-mouse vimentin antibody (1:200) at 4°C overnight. The cells were then incubated sequentially with fluorescently conjugated monkey anti-goat (1:500, Alexa Fluor 488 donkey anti-goat IgG, Life Technologies, Grand Island, NY) and goat anti-rabbit (1:500, Alexa Fluor 546 goat anti-rabbit IgG) secondary antibodies.

At 4 weeks after tumor transplantation, tumor tissues derived from the animals were sliced and embedded in paraffin. Immunohistochemical staining was performed using rabbit anti-mouse TNF-α (1:200), IL-6 (1:400) and Ki-67 (1:100) primary antibodies and goat anti-rabbit HRP-conjugated IgG (1:300), followed by staining of nuclei with hematoxylin.

### Statistical analysis

Data are presented as the means ± SD. Statistical analyses were performed using SPSS software, version 18.0. Differences between two groups were analyzed by Student's *t* test. The survival curves were determined using the Kaplan-Meier method and estimated with the log-rank test. *P* < 0.05 was considered statistically significant.

## SUPPLEMENTARY FIGURES


