# Supplementary material for: Diabetes mellitus stimulates pancreatic cancer growth and epithelial-mesenchymal transition-mediated metastasis via a p38 MAPK pathway
Source: Oncotarget. 2016 May 21;7(25):38539–50. doi: 10.18632/oncotarget.9533 (PMC5122409; doi:10.18632/oncotarget.9533)
Supplement: Supplementary file 1 [file oncotarget-07-38539-s001.pdf]

Diabetes mellitus stimulates pancreatic cancer growth and epithelial-mesenchymal transition-mediated metastasis via a p38 MAPK pathway

SUPPLEMENTARY FIGURES

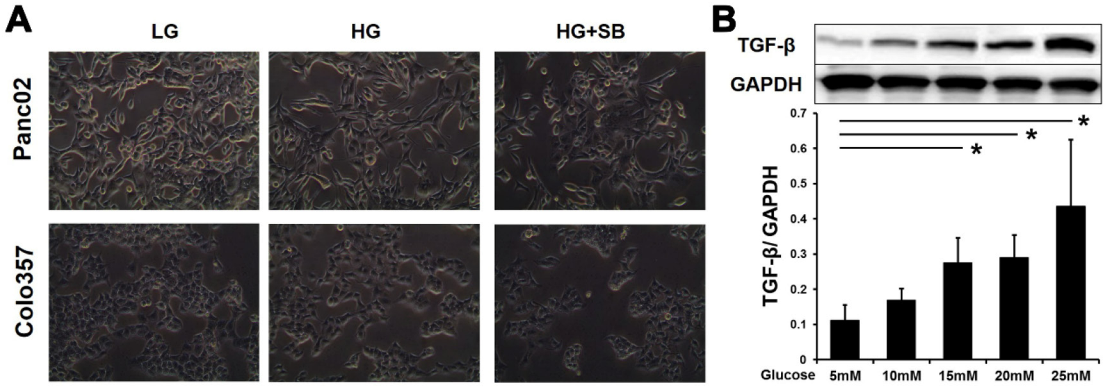

Supplementary Figure S1: A. Morphological changes in PC cells after culture in low-glucose (LG) medium, high-glucose (HG) medium, or HG medium containing 10 µM SB303580 for 24 h (200×). **B.** TGF-β expression levels in Panc02 cells after culture in medium with various glucose concentrations (5-25 mM) or HG medium (25 mM) containing 10 µM SB303580 for 24 h (n =3/group). * $P < 0.05$.

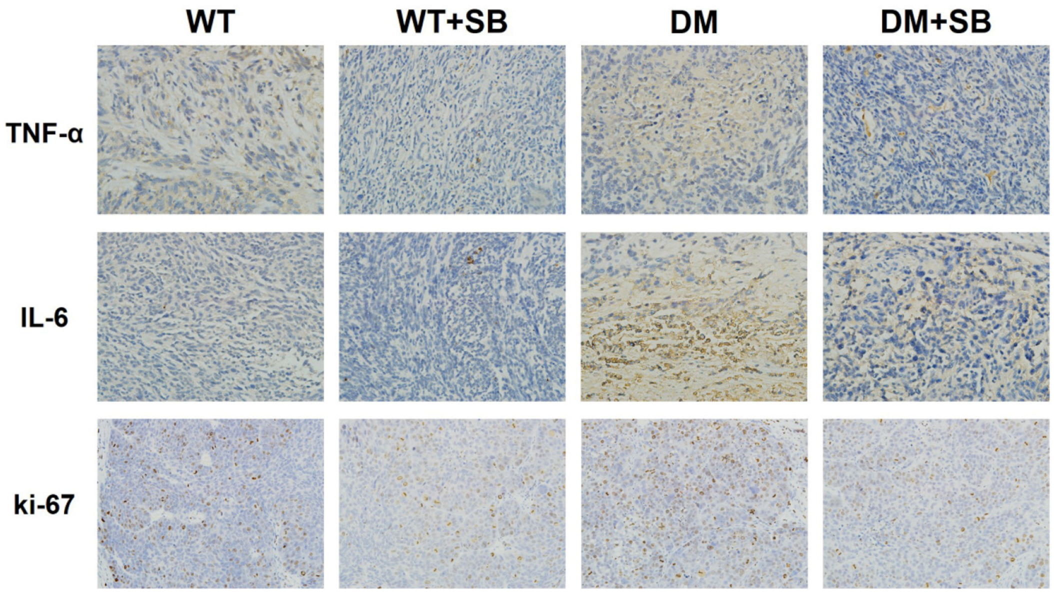

Supplementary Figure S2: Immunohistochemical analysis of TNF-α, IL-6 and Ki-67 expression in primary tumors from the WT, WT+SB, DM and DM+SB groups.
